# A Peek into Tropomyosin Binding and Unfolding on the Actin Filament

**DOI:** 10.1371/journal.pone.0006336

**Published:** 2009-07-24

**Authors:** Abhishek Singh, Sarah E. Hitchcock-DeGregori

**Affiliations:** 1 Department of Neuroscience and Cell Biology, Robert Wood Johnson Medical School, Piscataway, New Jersey, United States of America; 2 MD/PhD Program, Robert Wood Johnson Medical School, Piscataway, New Jersey, United States of America; 3 Joint Graduate Program in Biochemistry and Molecular Biology, University of Medicine and Dentistry, New Jersey Graduate School of Biomedical Sciences and Rutgers University, Piscataway, New Jersey, United States of America; Griffith University, Australia

## Abstract

**Background:**

Tropomyosin is a prototypical coiled coil along its length with subtle variations in structure that allow interactions with actin and other proteins. Actin binding globally stabilizes tropomyosin. Tropomyosin-actin interaction occurs periodically along the length of tropomyosin. However, it is not well understood how tropomyosin binds actin.

**Principal Findings:**

Tropomyosin's periodic binding sites make differential contributions to two components of actin binding, cooperativity and affinity, and can be classified as primary or secondary sites. We show through mutagenesis and analysis of recombinant striated muscle α-tropomyosins that primary actin binding sites have a destabilizing coiled-coil interface, typically alanine-rich, embedded within a non-interface recognition sequence. Introduction of an Ala cluster in place of the native, more stable interface in period 2 and/or period 3 sites (of seven) increased the affinity or cooperativity of actin binding, analysed by cosedimentation and differential scanning calorimetry. Replacement of period 3 with period 5 sequence, an unstable region of known importance for cooperative actin binding, increased the cooperativity of binding. Introduction of the fluorescent probe, pyrene, near the mutation sites in periods 2 and 3 reported local instability, stabilization by actin binding, and local unfolding before or coincident with dissociation from actin (measured using light scattering), and chain dissociation (analyzed using circular dichroism).

**Conclusions:**

This, and previous work, suggests that regions of tropomyosin involved in binding actin have non-interface residues specific for interaction with actin and an unstable interface that is locally stabilized upon binding. The destabilized interface allows residues on the coiled-coil surface to obtain an optimal conformation for interaction with actin by increasing the number of local substates that the side chains can sample. We suggest that local disorder is a property typical of coiled coil binding sites and proteins that have multiple binding partners, of which tropomyosin is one type.

## Introduction

The α-helical coiled coil is a ubiquitous protein folding and assembly motif present in proteins that participate in a wide variety of cellular functions, including muscle contraction, transcription, membrane trafficking, and metabolism [1]. The motif typically consists of two to four α-helical chains that associate into a left-handed helix to form a supercoil. The distortion due to the helical twist leads to underwinding of the coiled coil resulting in a heptad repeat every two helical turns [2]. In this pattern, the residues in the heptad repeat are labeled *a-b-c-d-e-f-g*. The *a* and *d* residues are frequently hydrophobic and pack at the interface, while the *e* and *g* residues are often oppositely charged and stabilize the coiled coil through interchain electrostatic interactions. Residues in the *b, c* and *f* positions are available for binding other proteins.

Tropomyosin (TM), the prototypical two-chained, parallel coiled coil, binds and stabilizes actin filaments in muscle and nonmuscle cells [3]. Tropomyosin's role is best established in striated muscle where it cooperatively regulates contraction in response to Ca^2+^ binding to troponin and myosin binding to actin [4]. Although the structure of tropomyosin has been determined using NMR and X-ray crystallography, it is less well understood how tropomyosin binds to its primary binding partner, actin [5]–[11]. Analysis of the sequence reveals a seven-fold repeat that has been proposed to represent seven quasi-equivalent sites for binding seven actins along the length of one tropomyosin molecule [12], [13]. McLachlan and Stewart noticed a pattern of alternating α and βrepeats; the Phillips pattern of seven charged and non-polar residues (referred to here as “consensus” residues) takes into account the coiled-coil structure and approximately corresponds to the α-repeats.

McLachlan and Stewart also noted a periodic distribution of small nonpolar amino acids, such as Ala at the coiled-coil interface that allows flexibility or bending of the tropomyosin supercoil to conform to the helical actin filament surface [14], [15]. The Ala or destabilizing clusters do not always correspond to the seven periodic repeats [8], [16]. Our previous work provides evidence that the flexibility or instability allowed by the small nonpolar amino acids at the interface, specifically the Ala clusters, is required for tropomyosin to bind to actin. In addition, we showed that co-localization of the consensus residues with a poorly packed, unstable interface is critical for optimal high affinity, cooperative binding [17]–[19]. The experiments provide support for the proposal that the periodic pattern of surface residues forms a recognition site for an actin subunit in the filament.

Among the seven quasi-equivalent periods in 284-residue tropomyosins (P1-P7), a series of mutagenesis experiments has shown that those with destabilizing Ala clusters (P1 and P5) are the most critical for actin binding. We refer to them as “primary” sites. The other periods contribute less to the overall actin affinity and here are called “secondary” sites. We have suggested that the secondary sites may bind to and modulate interaction with actin following initial binding via one of the primary sites [19], [20].

In the following work we ask the question: Can destabilization of a secondary site lead to an increase in the affinity of tropomyosin for actin? We hypothesize that a secondary site can be transformed into a primary site by the introduction of a destabilizing interface that allows the non-interface residues to obtain an optimal conformation to interact with actin by increasing the number of local substates that the amino acid functional groups can sample. We show that all actin binding sites are not created equal and that there are two components to binding, cooperativity and affinity. We suggest that the composition, location, and distance between actin binding periods in the tropomyosin molecule contribute to the components of binding in different ways. We infer from these results, and others, that the interaction of coiled coils with target molecules requires a locally flexible or slightly disordered coiled coil.

## Results

In the work presented here we have tested the hypothesis that destabilization of the interface of a secondary site should increase actin affinity. The coiled coil interface in site 2 (P2) or site 3 (P3), though not canonical, is more stable than in site P1 or P5. In P2 and P3 the Ala clusters are located between the consensus regions. Even though deletion of P2 or P3 results in a modest reduction in actin affinity, replacement of the Ala cluster with a canonical coiled coil interface dramatically reduces actin affinity [17], suggesting destabilization is essential for actin binding whether by direct participation of the P2-P3 region or by determining the overall molecular shape for binding the actin filament.

**Figure 1:**
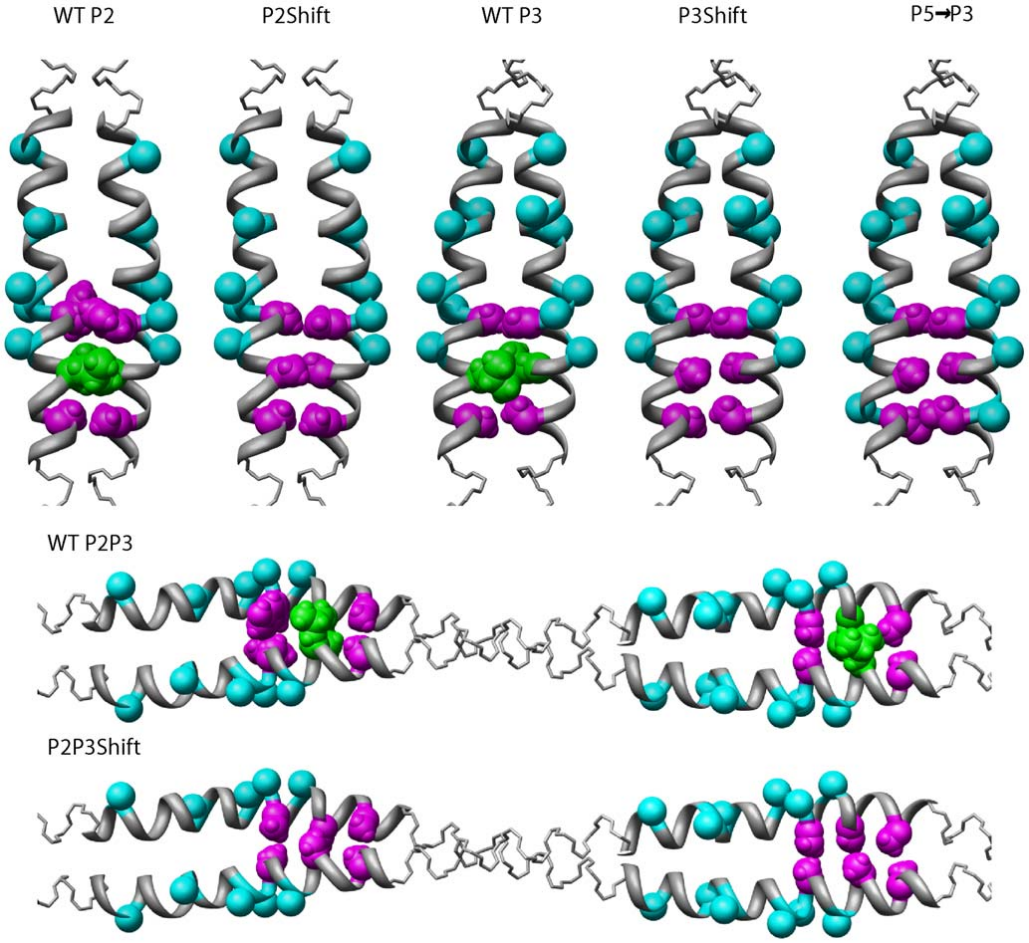
Models illustrating the P2 and P3 regions in wildtype and mutant tropomyosins. The side chains of the alanine clusters (magenta) and consensus actin binding sites (cyan) [13] are illustrated on a ribbon model of the 7 Å structure (pdb ID: 1C1G; [60]). Period 2, residues 46–69, and period 3, residues 88–111, are enlarged with the side chains of interface Ala residues (in magenta, space filling), canonical interface residues (green) and consensus residues (cyan, C_β_ in spacefill). The colors used are the same as in Table 1.

We designed mutants to transform secondary sites (P2, P3) into primary sites by introducing an Ala cluster in place of the native, more stable interface. To do this an Ala was “shifted” into the consensus region to mimic the Ala clusters at the P1 and P5 interfaces, creating the P2Shift and P3Shift mutants (Figure 1, Table 1; P2Shift = Y60A/L64A; P3Shift = L106A/A120L). In P3Shift we introduced A120L mutation C-terminal to P3 as a compensatory mutation since P3 is adjacent to the least stable region of the molecule (residues 130–190). In addition, we created P2P3Shift, combining the above mutations to create four primary sites. Based on studies with model peptides, the Ala substitutions in the context of P2 and P3 would destabilize the coiled coil [16], [21].

**Table 1:** Tropomyosin period 2 and period 3 cluster and period shift mutants.*

	d	e	f	g	a	b	c	d	e	f	g	a	b	c	d	e	f	g	a	b	c	d	e	f
	46	47	*48*	49	50	51	*52*	53	*54*	55	56	57	*58*	*59*	60	61	*62*	63	64	65	*66*	67	68	69
P2 wildtype	L	q	*K*	k	l	k	G	t	*E*	d	e	l	*D*	*K*	*y*	s	*E*	a	*l*	k	D	*a*	q	e
P2Shift															*a*				*A*			*a*		
Proposed			*K*										*E*	*K*			*D*							
Consensus			*or*				*NP*		*E*				*or*	*or*			*or*				*NP*			
Residues			*R*										*D*	*R*			*E*							

	d	e	*f*	g	a	b	*c*	d	*e*	f	g	a	*b*	*c*	*d*	e	*F*	g	*a*	b	*c*	*d*	e	f
	88	89	90	91	92	93	94	95	96	97	98	99	100	101	102	103	104	105	106	107	108	109	110	111
P3 wildtype	L	n	*R*	r	i	q	*L*	v	*E*	e	e	l	*D*	*R*	*a*	q	*E*	r	*L*	a	T	*a*	l	q
P3Shift															*a*				*a*			*a*		
P5→P3	v	a	*R*	k	l	v	*I*	i	*E*	s	d	l	*E*	*R*	*a*	e	*E*	r	*a*	e	*L*	*s*	e	s

* Residues in capitals are in proposed consensus actin binding positions [13]. Residues in capitals and italics are consensus acting binding residues. Residues in lower case and italics are at the interface at the Ala cluster positions [8]. See Figure 1 to correlate with the structure.

In a second strategy, we replaced P3 with P5, an established primary actin binding site (P5→P3) [18], [20]. We previously reported that P2 could replace P5, and that in the P5 position the P2 sequence with a shifted Ala cluster had higher affinity than with the native P2 interface [19]. In the present study we replaced the P3 sequence (residues 88–111) with that of P5 that contains an interface Ala cluster (residues 165–188; Table 1). In both P3Shift and P5→P3 the tropomyosins have alternating primary sites (P1, P3, P5). All mutations were made in rat striated muscle α-tropomyosin cDNA, and expressed in *E. coli* with an unacetylated N-terminal Met (Materials and Methods).

### The mutations locally destabilize tropomyosin with minimal effects on global stability

The aim of the P2Shift and P3Shift mutant design was to create destabilizing Ala clusters at the consensus sites with minimal effect on the overall stability. To evaluate the thermal stability of tropomyosin we used circular dichroism (CD) and differential scanning calorimetry (DSC).

**Figure 2:**
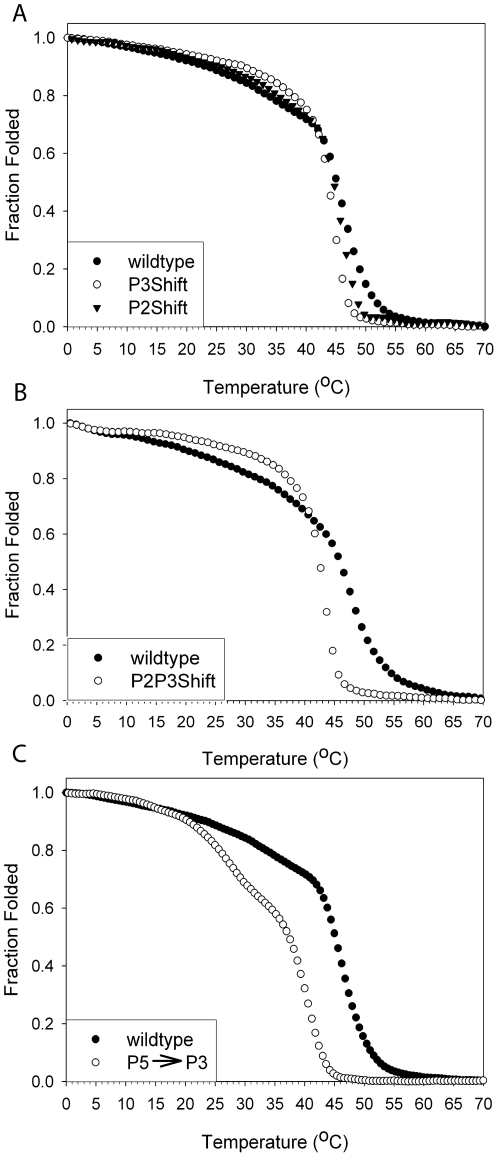
The effect on the thermal stability of the cluster and period replacement mutants. Fraction folded as measured by relative ellipticity at 222 nm as a function of temperature in 500 mM NaCl, 10 mM sodium phosphate pH 7.5, 1 mM EDTA, 0.5 mM DTT. The tropomyosin concentration was 0.01 mg/ml (1.6 µM). The T_M_s are reported in Tables 2 and 3. The fraction folded is relative to the mean residue ellipticity at 0°C where the proteins were fully folded. A. Symbols: •, wildtype; ○, P3Shift, ▾, P2Shift. B. Symbols: •, wildtype; ○, P2P3Shift. C. Symbols: •, wildtype; ○, P5→P3.

**Figure 3:**
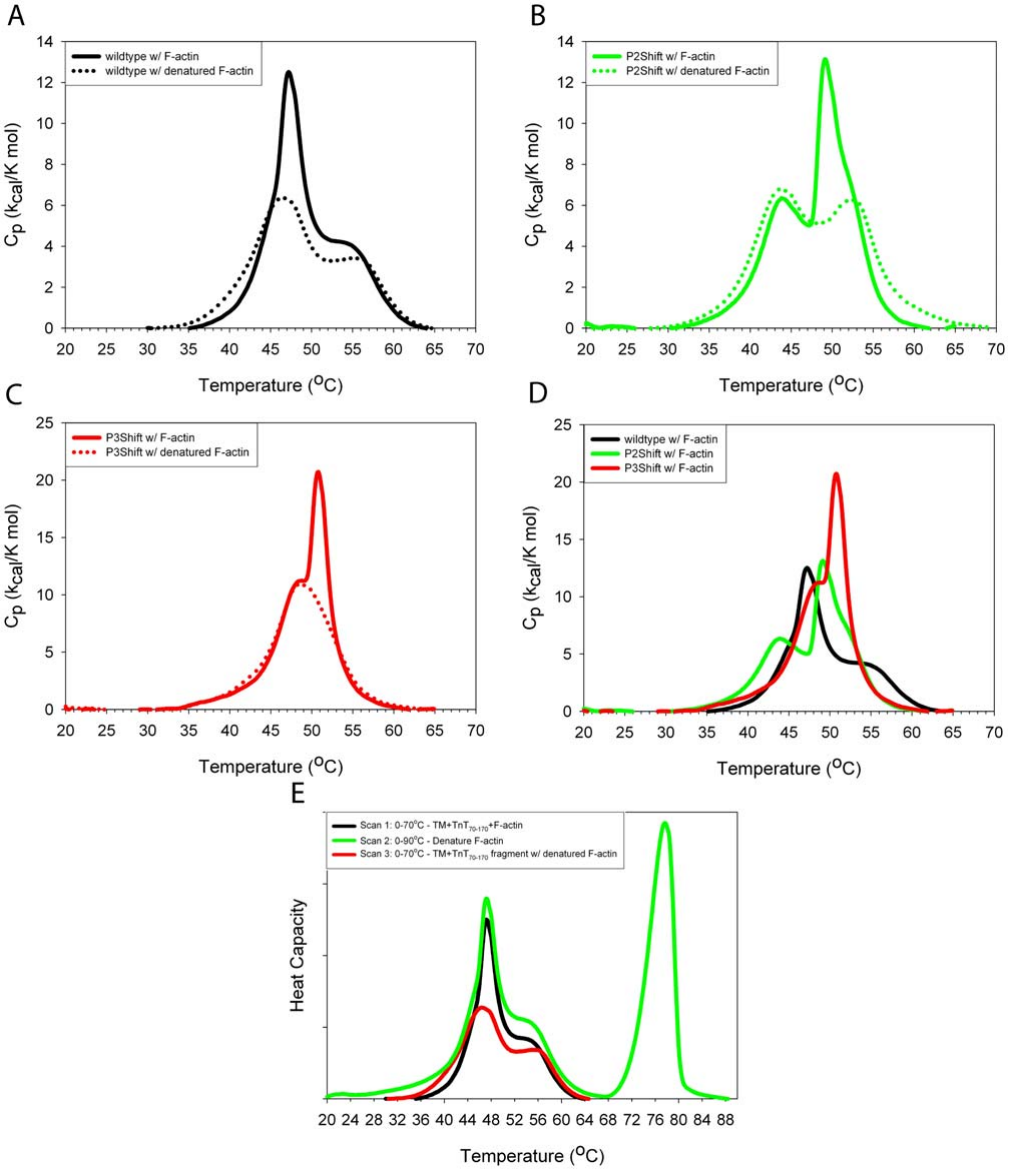
DSC scans of single cluster shift mutants with TnT_70–170_ in the presence and absence of F-actin. Tropomyosin (15 µM) and TnT_70–170_ (18 µM) were mixed with phalloidin (36 µM) stabilized F-actin (24 µM) in 100 mM NaCl, 10 mM Hepes pH 7.0, 2 mM MgCl_2_, 1 mM DTT and heated as described in Materials and Methods. The second (with F-actin-solid lines) and third (post-F-actin denaturation-dotted lines, TM- TnT_70–170_) scans are shown. A. wildtype (black); B. P2Shift (green); C. P3Shift (red); D. Combination of A-C in the presence of F-actin with the color scheme as indicated in A-C. The P2Shift and P3Shift mutants bind F-actin with higher affinity than wildtype. E. Experimental procedure: An excess of unacetylated recombinant tropomyosins and the TnT_70–170_ fragment was added to phalloidin-stabilized F-actin and the sample was heated a total of three times. An excess was added to ensure maximal binding of tropomyosin. The experiments were done in the presence of TnT_70–170_ because unacetylated tropomyosin binds poorly to F-actin. First, the sample was heated from 0–70°C (black line) and then cooled. This curve shows the dissociation of tropomyosin from F-actin (main peak) and the unfolding of TM-TnT_70–170_ . Second, the sample was heated to 90°C (green line) to denature the F-actin and subsequently cooled. In the third scan (magenta line), post-F-actin denaturation, the sample was heated to 70°C and only the TM+TnT_70–170_ signal is present. The first two scans are similar, and mark the tropomyosin unfolding/dissociation thermal peak, consistent with previously published results [22], [24], [62]. The thermal unfolding of tropomyosin is almost completely reversible, while F-actin denaturation is irreversible.

Introduction of an Ala cluster into P2 or P3 had little effect on the global stability of the molecule, as indicated by the slight decrease in overall T_M_ from 45.0°C to 44.5 and 43.4 °C, respectively (Figure 2A, Table 2). The thermal unfolding of wildtype tropomyosin analyzed using DSC showed the expected two major endotherms (Figure 3A, dotted line, Table 2). In this biphasic unfolding the first endotherm corresponds to the unstable middle region of the molecule including P5 (residues 130–190) and the C-terminal half whereas the second endotherm, 56.0°C in wildtype, is the unfolding of the N-terminal ∼half. In P2Shift both transitions occurred at lower temperatures (Figure 3B, dotted line). The unfolding of P3Shift was more cooperative and lacks the pretransition evident in the melts of the other forms, known to reflect the unfolding of the unstable middle of the molecule (Figure 3C, dotted line). The compensatory A120L mutation in P3Shift may stabilize this region and result in the single, lower temperature transition (49.0°C) with DSC, indicative of increased cooperativity of unfolding. P2P3Shift had similar characteristics and lower stability (Figure 2B, Table 2). The P5→P3 mutant was much less stable than the others; the initial transition reflects the low stability of the P5 region and its effect on the adjacent middle of the molecule (Figure 2C, Table 2). The mutant does not have the A120L mutation that adds stability to the P3Shift mutant. The DSC experiments were carried out in the presence of TnT_70–170_ (for the experiments with actin, see below), which has little effect on the thermal peaks of tropomyosin, consistent with previous results with full-length TnT (results not shown; [22]).

**Table 2:** The actin affinity and T_M_ from the ellipticity at 222 nm and DSC melts.

Tropomyosin	*K_app_* (×10^6^ M^−1^)^a^	α^H^ a	DSC T_M1_ (°C) b	DSC T_M2_ (°C) b	DSC TM Dissociation T_M3_ (°C) b	DSC *ΔΔH* (k_cal_/mol)^b^	CD T_M_ (°C) c
Wildtype	1.33±0.02	4.50±0.37	46.4	56.0	47.2	13.3	45.0
P2Shift	1.78±0.05	4.33±0.46	43.8	52.2	48.8	22.1	44.5
P3Shift	1.20±0.02	6.23±0.86	49.0		50.8	23.1	43.4
P2P3Shift	1.83±0.07	3.62±0.43	43.7		44.4	14.4	42.5
P5→P3	1.33±0.02	6.5±0.68	broad		37.6	37.6	37.4

a The values for *K_app_*, shown with standard errors. The data were fit to the Hill equation, and the *K_app_* and α^H^, Hill coefficient, are those reported by SigmaPlot (Figure 6).

b T_M1_ and T_M2_ represent the tropomyosin transitions obtained in the absence of F-actin. T_M3_ is the transition corresponding to tropomyosin dissociation from F-actin (Figure 3). The ΔΔH value was obtained by subtracting the total ΔH values of the endotherms in the presence and absence of F-actin. The difference is proportional to the amount of energy required for dissociation of tropomyosin bound F-actin.

c T_M_ is the point at which 50% of tropomyosin is unfolded (Figure 2). The experimental conditions differ for the three analyses and are described in the Materials and Methods as well as the figure legends. The values can be compared on a relative rather than absolute basis.

**Table 3:** The T_M_s of the light scattering, PIA excimer fluorescence, and unfolding.

Tropomyosin	Light Scattering T_M_ (°C) a	Excimer Peak (°C)^b^	CD T_M_ (°C) c
PIA Wildtype (Cys 190-PIA)	(45)	40 (37)	(45.0)
PIA P2Shift	(45)	41 (38)	(44.5)
PIA P3Shift	(46.5)	41 (40)	(43.4)
PIA C190S/L71C	(40.8)	41 (38)	35.9 (36.9)
PIA P2Shift C190S/L71C	(40.7)	40 (35)	35.4 (35.9)
PIA C190S/L113C	(40.6)	37 (broad)	36.4 (36.9)
PIA P3Shift C190S/L113C	(41.9)	38 (∼32)	36.0 (36.7)

a T_M_ is the value for half maximal dissociation of tropomyosin from F-actin or the 50% decrease in light scattering (Figures 4, 5).

b The value is the absolute maximum of the excimer peak in the presence of F-actin (Figures 4, 5).

c T_M_ is the point at which 50% of tropomyosin is unfolded (Figure 2). Values in parentheses are unlabeled tropomyosins, except in the case of the Excimer Peak column in which values in ( ) are in the absence of F-actin.

In addition to DSC, we analyzed local stability by introducing the fluorescent probe, pyrene iodoacetamide (PIA) near the mutation. When attached to Cys190 in striated muscle tropomyosin, the increase in pyrene excimer fluorescence has proven to be a sensitive monitor of the local unfolding of the middle of the tropomyosin molecule, including P5 [23]. During thermal denaturation this region unfolds prior to the main transition and is followed by the loss of excimer fluorescence when the chains separate. Mutations that encode a Cys for modification in P2 and P3 were introduced in P2Shift (L71C), P3Shift (L113C) and wildtype, as a control, to mimic the relationship of the naturally-occurring Cys190 to P5. The endogenous Cys190 was mutated to Ser to prevent labeling at this position.

The P2 and P3 mutations had little effect on the local unfolding in the region of Cys190; the temperature of the excimer peak of P3Shift was slightly increased, presumably reflecting the A120L mutation (Figure 4, dotted lines, Table 3). In contrast, with the probe at residue 71, adjacent to P2, the temperature of the excimer peak was reduced with the P2Shift mutation, compared to wildtype, reporting the destabilization of this region (Figure 5A, B dotted line, Table 3). Modification of residue 113 with pyrene destabilized the wildtype illustrated by a broad loss of excimer (Figure 5C, dotted line). There was an excimer peak in the presence of the P3Shift mutations (Figure 5D, dotted line), presumably reflecting the stabilizing effect of the A120L mutation. The shift mutations locally destabilize the coiled coil interface, as indicated by the higher or narrower excimer peak compared to the controls (Figure 5B,D). The distinct behavior of the probe in the three locations illustrates sensitivity to local conformational states of tropomyosin. In each case the temperature of the excimer peak was below the major unfolding transition or overall T_M_ of tropomyosin, indicating local unfolding.

**Figure 4:**
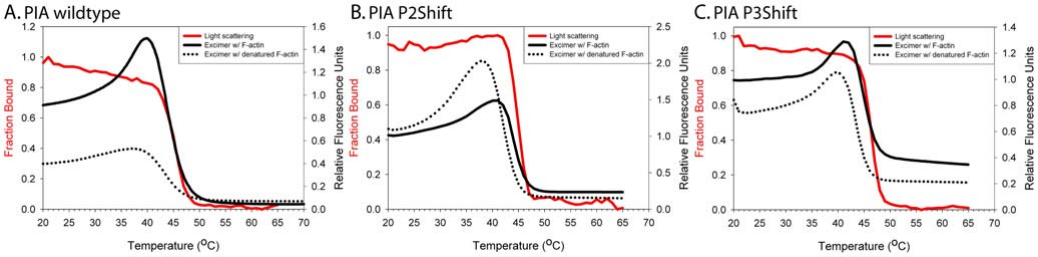
Excimer fluorescence of PIA-labeled (at Cys190) and light scattering of unlabeled wildtype, P2Shift, and P3Shift mutants. A. PIA wt; B. PIA P2Shift; C. PIA P3Shift. Excimer fluorescence and light scattering experiments: Tropomyosin (6 µM) and TnT_70–170_ (9 µM) were mixed with phalloidin (27 µM) stabilized F-actin (18 µM). Light scattering (red solid line and left axis with red label) was conducted with unlabeled tropomyosin due to interference from excimer peak in light scattering experiments with labeled tropomyosin. Excimer fluorescence (right axis) in the presence of actin (black solid line) and absence of actin (black dotted lines). Buffer conditions for all of the experiments: 100 mM NaCl, 10 mM sodium phosphate pH 7.5, 2 mM MgCl_2_, 1 mM DTT. Experimental Procedure: The excimer temperature scans were repeated three times at a 1°C/min scan rate [24]. First, the sample was heated from 0–70°C and then cooled again. Second, the sample was heated to 90°C to denature the F-actin and subsequently cooled. In the third scan, post F-actin denaturation, the sample was heated to 70°C and the excimer peak is detected in the absence of actin. The first two scans, in the presence of actin, were marked with appearance of the excimer peak at higher temperatures, indicating actin-induced stabilization.

**Figure 5:**
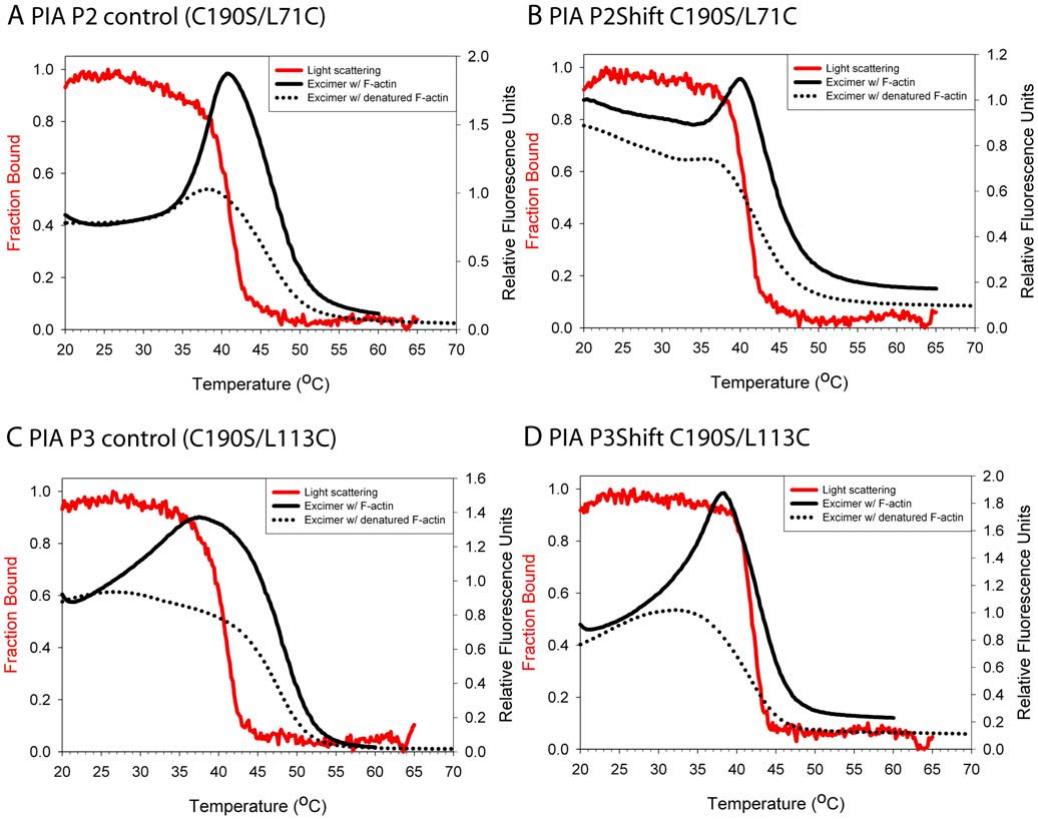
Excimer fluorescence of PIA-labeled and light scattering of unlabeled P2 and P3 mutants. A. P2 control (C190S/L71C) B. P2Shift C190S/L71C C. P3 control (C190S/L113C). D. P3Shift C190S/L113C. Light scattering (red solid line and left axis with red label) was conducted with unlabeled tropomyosin due to interference from excimer peak in light scattering experiments with labeled tropomyosin. Excimer fluorescence (right axis) in the presence of actin (black solid line) and absence of actin (black dotted lines). Buffer conditions and the experimental procedure are as described for Figure 4. The P2Shift and P3Shift mutations locally stabilize the interaction with actin.

### Functional analysis: Actin affinity

**Figure 6:**
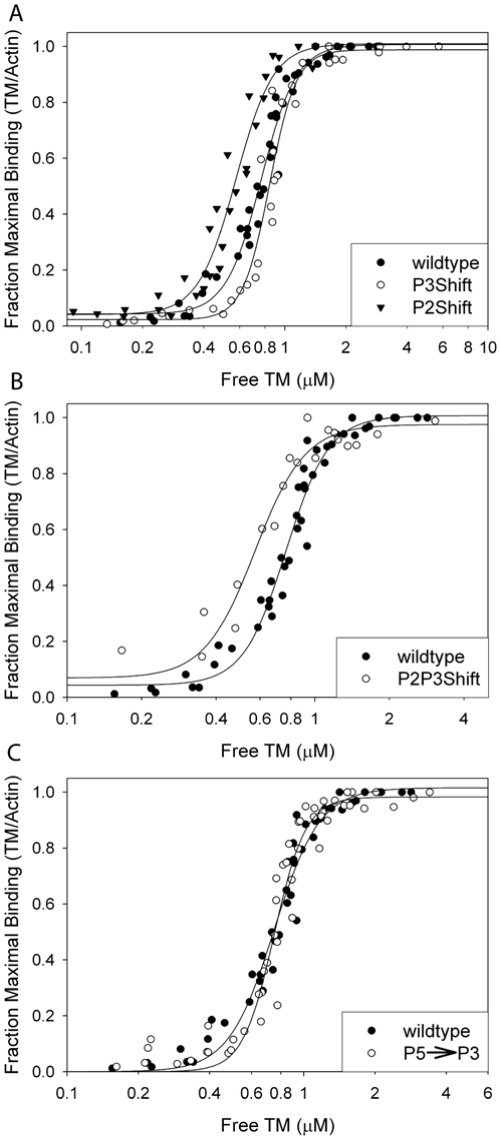
The effect the mutations on the actin affinity measured by cosedimentation with F-actin. Binding to filamentous actin. Tropomyosin (0.1–10 µM, depending on the tropomyosin) and 0.12–12 µM troponin T_70–170_ [44] were combined with 5 µM actin and sedimented at 20°C in 250 mM NaCl, 10 mM TrisHCl, pH 7.5, 2 mM MgCl_2_, and 0.5 mM DTT. Stoichiometric binding of 1 tropomyosin: 7 actins is represented by the 1.0 fraction of maximal binding. The apparent K_app_s are reported in Table 2. A. Symbols: •, wildtype; ○, P3Shift, ▾, P2Shift. B. Symbols: •, wildtype; ○, P2P3Shift. C. Symbols: •, wildtype; ○, P5→P3. The mutations affect the affinity and cooperativity of binding.

The actin affinity of the mutant tropomyosins was evaluated by cosedimentation with F-actin (Figure 6, Table 2) and differential scanning calorimetry (DSC) (Figure 3, Table 2). All four mutants bound F-actin, but differed in the affinity and cooperativity of binding. Introduction of the P2Shift mutations increased actin affinity whereas the P3Shift mutant bound with greater cooperativity than wildtype, but slightly reduced affinity (Figure 6A). The effects of the mutations when combined in the P2P3Shift mutant were not additive (Figure 6B). The affinity was greater (similar to P2) but without increasing the cooperativity of binding. When P3 was replaced with P5 (P5→P3), the cooperativity increased, but the affinity was unchanged (Figure 6C).

The DSC studies with tropomyosin bound to actin allow thermodynamic analysis of the affinity and cooperativity of actin binding of the P2Shift and P3Shift mutants as well as the effect of binding on the thermal unfolding of tropomyosin. The interaction of tropomyosin with phalloidin-stabilized F-actin results in the appearance of a new endothermic peak in the DSC, compared to tropomyosin alone. This peak correlates with the unfolding of tropomyosin bound to F-actin accompanied by dissociation, as proposed for reversible unfolding of oligomeric proteins [24]–[26].

The DSC denaturation experiments indicate that the P2Shift and P3Shift mutants bound tighter to F-actin than wildtype (major peaks in Figure 3A-C, solid lines, 3D; Table 2). Both mutants exhibited higher unfolding/dissociation peak temperatures and greater *ΔΔH* values than wildtype. The *ΔΔH* is the total enthalpy in the presence of actin, including the tropomyosin unfolding on actin, minus the TM + TnT_70–170_ with no actin. The *ΔΔH* is a measure of the difference energy required to dissociate tropomyosin from actin. Compared to wildtype values, an increase in *ΔΔH* coupled with an increase in T_M_ of unfolding of tropomyosin from actin is proportional to an increase in the binding constant. The width of the actin-bound tropomyosin thermal transition indicates the cooperativity of unfolding. The greater cooperativity of P3Shift on actin seen in the cosedimentation experiments is supported here by the narrower thermal transition, compared to wildtype.

### Local unfolding of tropomyosin on the actin filament

The DSC experiments showed that tropomyosin dissociates from F-actin in a single endothermic peak. However, it is unclear how each region of tropomyosin contributes to the unfolding in the presence of actin, and how unfolding relates to dissociation from actin and to chain separation. If the regions of tropomyosin involved in actin binding become ordered upon binding to the filament, a local probe should report local stabilization upon binding and destabilization prior to dissociation from the filament and chain separation. To address this, we return to the pyrene iodoacetamide-modified (PIA) tropomyosins we used to analyze local unfolding during tropomyosin denaturation.

In these experiments the PIA-tropomyosins were combined with phalloidin-stabilized actin, as for the DSC experiments. Tropomyosin bound to the actin filament will be locally constrained via binding interactions leading to poor excimer formation. Temperature-dependent dissociation of the labeled region of tropomyosin from F-actin allows the pyrene labels to reorient for excimer formation [27]. Correlation of this data with light scattering provides insight into the sequence of local unfolding in relation to chain separation and dissociation from the actin filament.

The pyrene excimer fluorescence (Figure 4, solid black line) was measured as a function of temperature and compared to that of PIA-tropomyosin in the absence of actin (dotted line). Light scattering increases when tropomyosin binds F-actin and can be used as an indicator of binding [24], [28]. Tropomyosin alone (and with TnT_70–170_) has minimal light scattering. Dissociation of the F-actin-TM-TnT complex is reversible as the light scattering intensity decreases with heating and increases with cooling. The temperature of dissociation of the complex, i.e. the temperature at which there is a 50% decrease in light scattering, correlates with the dissociation peak observed using DSC at similar concentrations and ratios of tropomyosin to F-actin. Therefore, the unfolding of actin-bound tropomyosin is accompanied by its dissociation from actin [29].

We discussed above how pyrene probes introduced at three different regions of tropomyosin (P2, P3, and P5) report region-specific unfolding. All locally unfold prior to or coincident with chain separation. The results in Figures 4 and 5 show that in all cases the pyrene excimer fluorescence peak occurred at a higher temperature when tropomyosin is bound to actin than when it is free, indicating that the region with the probe is stabilized by interaction with F-actin. Second, the rise (local unfolding) and decrease (chain dissociation) in excimer fluorescence preceded or coincided with dissociation from phalloidin-stabilized F-actin. The shift mutations locally stabilize the interaction with actin as evidenced by the greater increase in the temperature of the excimer peak upon actin binding or increased cooperativity of chain dissociation (Figure 5, compare A with B and C with D, Table 3). The increase in excimer fluorescence may be due to local unfolding of tropomyosin on the F-actin or to local dissociation from actin. In cases where the excimer fluorescence increases prior to dissociation from F-actin (as indicated by light scattering), the effect may result from dynamic local chain fluctuations while other parts of tropomyosin remain in contact with F-actin.

Since the mutations introduced for PIA modification, and the modifications themselves influence stability and actin affinity, it is not possible to compare directly the P2, P3 and P5 regions to each other, or to gain insight into sequential unfolding of the periods. We can conclude, however, that specific regions of tropomyosin are locally and possibly individually stabilized upon binding F-actin, and that they unfold prior to chain separation and dissociation from actin. Destabilization of P2 and P3 increases the local and overall affinity of tropomyosin for actin.

## Discussion

Interruptions in tropomyosin's canonical coiled coil interface with clusters of destabilizing residues such as Ala have been proposed to have a role in providing the flexibility needed to assume a complementary form to that of the helical actin filament [6], [8], [30], [31]. Here we report that the destabilizing Ala clusters, depending on their location in the sequence, can also influence cooperative actin binding in a positive fashion. We further show that the unstable regions are locally stabilized upon binding actin, and that they unfold in multiple overlapping units prior to the molecule's dissociation from actin and cooperative chain separation. The regions of instability, especially those that are not embedded in actin binding sites, may allow the supercoil to assume the conformation required for binding to the actin helix, and be responsible for the variable length of the supercoil. However, we suggest that in certain instances the destabilization is critical for direct binding of tropomyosin to actin subunits and for its cooperative regulation of the actin filament.

The cooperative binding of tropomyosin to F-actin has a K_app_ in the 10^6^ to 10^7^ M^−1^ range, depending on the isoform and ionic conditions. However, the affinity of individual tropomyosin molecules is weak, with a K_a_ of only 2–3×10^3^ M^−1^ [32]–[34], and that of the seven individual actin binding sites much less, if their contributions are equivalent. In order for tropomyosin to shift cooperatively its azimuthal position on the actin filament in response to myosin binding and Ca^2+^ binding to troponin C, it is essential that interactions along the filament be weak, and of varying strength, and that there be “communication” along the filament. The present work, along with published studies, documents a requirement for residue specificity in non-interface residues and shows that the actin binding sites are distinctive in their contributions to the overall affinity, as well as to the cooperativity of binding [18]–[20].

In the native sequence only period 1 (P1) and period 5 (P5) of the seven proposed periodic sites have destabilizing Ala clusters within the consensus region; deletion mutagenesis has shown these regions contribute the most to cooperative actin binding [20], [35]. Detailed analysis of P5 showed that specific surface residues and a destabilizing interface are both required for actin binding [18], [19]. We proposed that local interface instability or flexibility allows tropomyosin to obtain an optimal conformation to interact with actin.

In contrast, P2 and P3, located in the most stable region of tropomyosin, have stabilizing interfaces. Deletion of P2 and P3, alone or together, or replacement of the first 21 residues of each period with a GCN4 leucine zipper sequence reduced actin affinity but did not destroy binding [32], [34], [36]. The results suggest that, unlike the ends or P5 that are of primary importance, P2 and P3 contribute less to the overall actin affinity of the native molecule and may be considered secondary sites. The present work shows that transfer of a destabilizing Ala cluster to a location within the consensus site of P2 or P3, or replacement of P3 with the homologous P5 sequence that contains an Ala cluster, is sufficient to increase the affinity of tropomyosin for actin, thus transforming a secondary site into a primary site, as shown by cosedimentation and DSC in the presence of F-actin.

Tropomyosin, despite having an uninterrupted heptapeptide repeat of hydrophobic amino acids diagnostic of coiled coils, is a dynamic, subtly-designed protein. Proteolytic and spectroscopic analyses of tropomyosin have established that the central region of the molecule that includes P4 and P5, residues ∼130–190, is locally unstable and at least partially unfolded at physiological temperature [37]–[39]. This region is critical for binding actin as well as the troponin core domain, and for Ca^2+^ dependent regulation of the thin filament with troponin [18], [19], [34], [35], [40], [41]. Mutagenesis studies of the region containing residues 165–185 have shown that an unstable interface in this region is essential for actin binding and its participation as a primary actin binding site [18], [19].

In the present work, pyrene iodoacetamide probes introduced adjacent to the destabilized P2 and P3 sites to probe conformation reported local unfolding of the coiled coil at physiological temperatures, as for P5. Binding to actin stabilized these regions, as previously shown for the unstable middle region of the molecule [29], [42], a general feature of actin binding [24], [43]. We propose that local disorder or instability is a general requirement for the regions of tropomyosin involved in binding to actin, or other target proteins [44]. Increasingly there have been reports of local disorder encoded into protein sequences and structures [45], [46]. It is possible that this property is typical of proteins that have multiple binding partners, i.e. signaling molecules, of which tropomyosin is one type.

Our present and previous work indicates that the tropomyosin coiled coil is locally destabilized at regions of the molecule that bind to actin and other proteins, and that these regions contribute to the overall affinity and cooperativity of binding to actin in a sequence-specific fashion. We propose that these regions demonstrate *flexible* specificity. The concept of flexible specificity refers to the ability of functionally important regions of tropomyosin to reorient to maximally interact with its binding partners.

Intrinsically disordered domains are recognized to be a wide-spread protein-protein recognition mechanism [46]. Coiled coils also utilize intrinsic disorder to interact with target proteins. In the bacterial type III secretion system, there is mounting evidence that the structure of these injectisomes alter as they bind their cognate folded protein targets due to the extreme flexibility and propensity for coiled-coil interactions of the injectisomes themselves [47], [48]. Although crystal structures show tropomyosin to be an uninterrupted coiled coil, it is well-established that the middle of the molecule is not coiled coil at physiological temperature, though the extent of disorder has not been defined. Intermolecular complexes between tropomyosin's ends have K_d_s of 0.5–20 µM and are in equilibrium between folded and unfolded conformations [5], [6], [49]. We suggest that in addition to coupling via the ends, the intrinsic instability of the actin binding domains, and the long-range interactions between them, underlie the mechanism of cooperative binding of tropomyosin to actin. An allosteric coupling model incorporating intrinsic disorder was recently published [50]. In the case of tropomyosin, interaction of primary sites with actin may provide initial places for tropomyosin to “touch down” and to help position weaker, secondary sites on the filament. The sequence by which tropomyosin's sites bind along the filament cannot be answered by our methods. Whether it initially binds via the ends, the middle, or both, we cannot say.

In a recent review Holmes and Lehman put forward the proposal that the intrinsic helical contour of tropomyosin complements the actin filament structure by having a shape that allows it to interact with all seven actins along its length [30]. They suggest that the bends observed at the Ala cluster sites contribute to the molecule's shape, as a sort of bias towards the actin filament. While the Ala clusters and other regions of instability likely do allow tropomyosin the quasi-segmental flexibility to conform to the actin filament helix, we argue it does so by local destabilization, rather than segmental bending [17]. In several of the crystal structures not all Ala clusters are associated with bends; the bends may result from inter-molecular packing.

Tropomyosin's ability to bind actin depends on the cooperative nature of its interaction. Here we show that the source of the cooperativity is not only end-to-end; internal periods may also contribute. Destabilization of the coiled coil interface in P3 or replacement with the P5 sequence increases the cooperativity of binding. It is also well-established that the cooperativity of tropomyosin binding to actin and regulation is unrelated to the extent of end-to-end association [51], [52], raising the question of mechanism: is the cooperativity through the ends, through actin, or along the tropomyosin itself? We can only raise this question to emphasize that this fundamental aspect of actin filament regulation remains unanswered.

## Materials and Methods

### DNA construction and protein purification

Striated muscle α-tropomyosin mutants were made using rat striated α-tropomyosin cDNA, provided by Dr. Bernardo Nadal-Ginard [53], cloned in pET11d [54] for expression in *E. coli*. The mutations were made using the oligonucleotides listed below (mutations in lower case and underlined) and their reverse complements using two stage PCR as previously described [17]. The mutations were verified by complete sequencing of the cDNA at the DNA Core Facility at UMDNJ.

Cluster Shift mutants:

Y60A/L64A (used for P2Shift and P2P3Shift) – 5′ - GATGAACTGGACAAAgcgTCCGAGGCTgcgAAAGATGCCCAGGAG – 3′

L106A (used for P3Shift and P2P3Shift) – 5′ – CGGGCTCAGGAGCGGgccGCCACAGCTCTACAG – 3′

A120L (for P3Shift and P2P3Shift) – 5′ – GAGGCTGAGAAGGCTctgGATGAGAGTGAGAGA – 3′

Period replacement mutant; residues 165–188 of P5 replace residues 88–111 of P3:

P5→P3 Part 1 – 5′ – GCTGACGTAGCATCTgTGGCgCGcAAaCTGGTgATtATtGAaAGcGATCTgGATCGGGCTCAGGAG – 3′

P5→P3 Part 2 – 5′ – CTGGTgATtATtGAaAGcGATCTggaaCGcGCGGAaGAGCGGCTGGCCACAGC - 3′

P5→P3 Part 3 – 5′ – CTggaaCGcGCGGAaGAGAGgGCgGAaCTgTCcGAAGGcAAGCTGGAGGAGGCTGAGAAGG – 3′

Cysteine mutants for pyrene experiments:

C190S (used for PIA C190S/L71C, PIA C190S/L113C, PIA P2Shift C190S/L71C, PIA P3Shift C190S/L113C) – 5′ – GAGCTCTCGGAAGGCAAAtctGCGGAGCTTGAAGAAGAG – 3′

L71C (used for PIA C190S/L71C and PIA P2Shift C190S/L71C) – 5′ – GATGCCCAGGAGAAAtgcGAGCTGGCCGAGAAAAAGG – 3′

L113C (used for PIA C190S/L113C and PIA P3Shift C190S/L113C)– 5′ – CCACAGCTCTACAGAAGtgcGAGGAGGCTGAGAAGGC – 3′

Mutants were expressed in *E. coli* BL21 (DE3) cells [54] and were purified as previously described [55] by ammonium sulfate fractionation and chromatography on DE52 cellulose and hydroxylapatite.

Actin was purified from acetone powder of pectoral chicken skeletal muscle actin [56]. The recombinant fragment of human cardiac TnT_70–170_ used in all of the binding experiments was previously described [44].

Tropomyosin concentration was determined by measuring the difference spectrum of tyrosine [57]. The concentration of actin was determined by the difference method using the exctinction coefficient of 2357 per Tyr (16 Tyr/actin) and 830 per Trp (4 Trp/actin) (Fasman 1989 Practical Handbook of Biochem and Mol Bio CRC Press). Finally the concentration of the cTnT_70–170_ fragment was determined by the biuret assay.

### Circular dichroism and fluorescence measurements

Thermal stability measurements were made by following the ellipticity of tropomyosin at 0.1 mg/ml at 222 nm as a function of temperatures in 0.5 M NaCl, 10 mM sodium phosphate pH 7.5, 1 mM EDTA, 0.5 mM DTT (unless otherwise noted) in an Aviv Model 215 and 400 spectropolarimeter (Lakewood, NJ) with a total fluorescence attachment. The observed T_M_ is defined as the temperature at which the ellipticity at 222 nm, normalized to a scale of 0 to 1, is equal to 0.5.

The tropomyosins were modified at Cys190 with pyrene iodoacetamide in the presence of GuHCl following the protocol of Ishii and Lehrer [23]. The bound pyrene was determined to be 80–110% based on the extinction coefficient of bound pyrene iodoacetamide and the free sulfhydryl determined using [NbS]_2_ [41].

Temperature dependence of PIA excimer formation in the presence and absence of F-actin was measured using an Aviv CD model 400 instrument with a fluorescence attachment. The excitation wavelength was 340 nm. Monomer peaks appear at 385 nm and 405 nm while the excimer peak is shifted to 480 nm. Excimer fluorescence was monitored using a 475 nm interference filter. For experimental details see Figure 4.

### Differential scanning calorimetry

Calorimetric measurements were performed on a Model 6100 Nano II differential scanning calorimeter (Calorimetry Sciences Corp., American Fork, UT) with 0.3 ml cells from 0°C to either 70°C or 90°C at a rate of 1°C/min, pressure of 2 atm, and a 1 mg/ml tropomyosin protein concentration in 100 mM NaCl, 20 Hepes pH 7.0, 2 mM MgCl_2_, and 1 mM DTT (unless otherwise noted). The reversibility of the thermal transitions was checked by three or more heating and cooling runs. The calorimetric traces were corrected for the instrumental background by subtracting a scan with buffer in both cells. All calculations were carried out using CpCalc, a Windows based program supplied by the DSC vendor and Origin software (MicroCal). A molecular weight of 66 kDa was used for coiled coil tropomyosin dimers.

### Actin binding assays

The affinity of tropomyosin for filamentous actin was measured by cosedimentation [55]. Tropomyosin (0.1–10 µM, depending on the tropomyosin) and 0.12–12 µM troponin T_70–170_ [44] were combined with 5 µM actin and co-sedimented at 20°C in 250 mM NaCl, 10 mM TrisHCl, pH 7.5, 2 mM MgCl_2_, and 0.5 mM DTT. The TnT_70–170_ fragment, which binds to the carboxyl terminus of tropomyosin [44], was added at a 1.2× molar excess to increase the affinity of unacetylated, recombinant striated muscle tropomyosin for actin. The pellets and supernatants were analyzed by SDS-PAGE, stained with Coomassie blue, and analyzed on a Molecular Dynamics model 300A computing densitometer (Sunnyvale, CA; [58], [59]). The binding constant K_app_ of tropomyosin for F-actin and the Hill coefficient (α^H^) were determined by fitting the experimental data to the following equation using SigmaPlot (Jandel Scientific, San Rafael, CA):
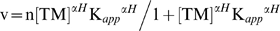
where v = fraction maximal tropomyosin binding to actin, n = maximal tropomyosin bound, [TM] = [TM]_free_. The TM:actin ratio was normalized to 1.0 by dividing the TM:actin ratio obtained from densitometry by the observed Tm:actin maximal ratio at saturation.

### Differential scanning calorimetry actin binding assay

In the tropomyosin-F-actin complex, tropomyosin and F-actin melt separately, and the thermal transitions of each can be distinguished despite a slight overlap. The interaction of tropomyosin with F-actin results in the appearance of a new endothermic peak in the DSC; this peak has been correlated to unfolding of actin-bound tropomyosin leading to dissociation from F-actin, as proposed for reversible unfolding of oligomeric proteins [24]–[26]. To achieve a better separation of the thermal transitions, F-actin was stabilized with phalloidin. In addition, thermal unfolding of tropomyosin is almost completely reversible, while F-actin denatures irreversibly [24]. Details of experimental procedure are in the legend of Figure 3.

### Correlation of light scattering and excimer fluorescence with tropomyosin dissociation from actin

Combination of both techniques allowed us to determine when tropomyosin dissociates from actin (light scattering) and to correlate that with unfolding of the P2, P3, or P5 regions on actin (excimer fluorescence). Light scattering experiments were carried out with a mixture of 6 µM tropomyosin, 12 µM TnT_70–170_, 18 µM F-actin, and 27 µM phalloidin. Light scattering experiments were followed by excimer fluorescence scans. The details of experimental procedure are in the legends to Figures 3 and 4.

The light scattering experiments were carried out in an Aviv model 400 circular dichroism instrument fitted with a total fluorescence attachment to measure light scattering at 90^o^. The 475 nm cutoff filter used to monitor pyrene excimer fluorescence was removed for the light scattering measurements. Light scattering measurements were conducted at 245 nm with unlabeled tropomyosin mutants to eliminate the confounding PIA signal. Light scattering temperature scans from 20°C to 65°C at a scan rate of 1°C/min were repeated at least two times on the same sample. F-actin alone was used for baseline correction. All calculations were done using Origin software (MicroCal). Each scattering curve was normalized to 1.0 by dividing the observed data by the maximal scattering.

### Molecular modeling

Modeling was carried out using SYBYL (Tripos, Inc.). The mutations were introduced into the 7 Angstrom structure [60]; PDB entry 1C1G). The molecular graphics images were produced using the UCSF Chimera package from the Computer Graphics Laboratory, University of California, San Francisco [61] supported by NIH P41 RR-01081.

## References

[1] Burkhard P, Stetefeld J, Strelkov SV (2001). Coiled coils: a highly versatile protein folding motif.. Trends Cell Biol.

[2] Crick FHC (1953). The packing of alpha-helices. Simple coiled-coils.. Acta Crystallographica.

[3] Gunning PW, Schevzov G, Kee AJ, Hardeman EC (2005). Tropomyosin isoforms: divining rods for actin cytoskeleton function.. Trends Cell Biol.

[4] Perry SV (2001). Vertebrate tropomyosin: distribution, properties and function.. J Muscle Res Cell Motil.

[5] Greenfield NJ, Kotlyanskaya L, Hitchcock-Degregori SE (2009). Structure of the N Terminus of a Nonmuscle alpha-Tropomyosin in Complex with the C Terminus: Implications for Actin Binding (dagger) (double dagger).. Biochemistry.

[6] Greenfield NJ, Huang YJ, Swapna GV, Bhattacharya A, Rapp B (2006). Solution NMR structure of the junction between tropomyosin molecules: implications for actin binding and regulation.. J Mol Biol.

[7] Phillips GN, Fillers JP, Cohen C (1986). Tropomyosin crystal structure and muscle regulation.. J Mol Biol.

[8] Brown JH, Kim KH, Jun G, Greenfield NJ, Dominguez R (2001). Deciphering the design of the tropomyosin molecule.. Proc Natl Acad Sci U S A.

[9] Brown JH, Zhou Z, Reshetnikova L, Robinson H, Yammani RD (2005). Structure of the mid-region of tropomyosin: bending and binding sites for actin.. Proc Natl Acad Sci U S A.

[10] Nitanai Y, Minakata S, Maeda K, Oda N, Maeda Y (2007). Crystal structures of tropomyosin: flexible coiled-coil.. Adv Exp Med Biol.

[11] Li Y, Mui S, Brown JH, Strand J, Reshetnikova L (2002). The crystal structure of the C-terminal fragment of striated-muscle alpha-tropomyosin reveals a key troponin T recognition site.. Proc Natl Acad Sci U S A.

[12] McLachlan AD, Stewart M (1976). The 14-fold periodicity in alpha-tropomyosin and the interaction with actin.. J Mol Biol.

[13] Phillips GN (1986). Construction of an atomic model for tropomyosin and implications for interactions with actin.. J Mol Biol.

[14] McLachlan AD, Stewart M, Smillie LB (1975). Sequence repeats in alpha-tropomyosin.. J Mol Biol.

[15] Parry DA (1975). Analysis of the primary sequence of alpha-tropomyosin from rabbit skeletal muscle.. J Mol Biol.

[16] Kwok SC, Hodges RS (2004). Stabilizing and destabilizing clusters in the hydrophobic core of long two-stranded alpha-helical coiled-coils.. J Biol Chem.

[17] Singh A, Hitchcock-DeGregori SE (2003). Local destabilization of the tropomyosin coiled coil gives the molecular flexibility required for actin binding.. Biochemistry.

[18] Singh A, Hitchcock-DeGregori SE (2006). Dual requirement for flexibility and specificity for binding of the coiled-coil tropomyosin to its target, actin.. Structure.

[19] Singh A, Hitchcock-DeGregori SE (2007). Tropomyosin's periods are quasi-equivalent for actin binding but have specific regulatory functions.. Biochemistry.

[20] Hitchcock-DeGregori SE, Song Y, Greenfield NJ (2002). Functions of tropomyosin's periodic repeats.. Biochemistry.

[21] Lu SM, Hodges RS (2004). Defining the minimum size of a hydrophobic cluster in two-stranded alpha-helical coiled-coils: effects on protein stability.. Protein Sci.

[22] Kremneva EV, Nikolaeva OP, Gusev NB, Levitsky DI (2003). Effects of troponin on thermal unfolding of actin-bound tropomyosin.. Biochemistry (Mosc).

[23] Ishii Y, Lehrer SS (1990). Excimer fluorescence of pyrenyliodoacetamide-labeled tropomyosin: a probe of the state of tropomyosin in reconstituted muscle thin filaments.. Biochemistry.

[24] Levitsky DI, Rostkova EV, Orlov VN, Nikolaeva OP, Moiseeva LN (2000). Complexes of smooth muscle tropomyosin with F-actin studied by differential scanning calorimetry.. Eur J Biochem.

[25] Johnson CR, Morin PE, Arrowsmith CH, Freire E (1995). Thermodynamic analysis of the structural stability of the tetrameric oligomerization domain of p53 tumor suppressor.. Biochemistry.

[26] Boudker O, Todd MJ, Freire E (1997). The structural stability of the co-chaperonin GroES.. J Mol Biol.

[27] Lehrer SS (1997). Intramolecular pyrene excimer fluorescence: a probe of proximity and protein conformational change.. Methods Enzymol.

[28] Wegner A (1979). Equilibrium of the actin-tropomyosin interaction.. J Mol Biol.

[29] Kremneva E, Boussouf S, Nikolaeva O, Maytum R, Geeves MA (2004). Effects of two familial hypertrophic cardiomyopathy mutations in alpha-tropomyosin, Asp175Asn and Glu180Gly, on the thermal unfolding of actin-bound tropomyosin.. Biophys J.

[30] Holmes KC, Lehman W (2008). Gestalt-binding of tropomyosin to actin filaments.. J Muscle Res Cell Motil.

[31] Brown JH, Cohen C (2005). Regulation of muscle contraction by tropomyosin and troponin: how structure illuminates function.. Adv Protein Chem.

[32] Hitchcock-DeGregori SE, Varnell TA (1990). Tropomyosin has discrete actin-binding sites with sevenfold and fourteenfold periodicities.. J Mol Biol.

[33] Wegner A, Walsh TP (1981). Interaction of tropomyosin-troponin with actin filaments.. Biochemistry.

[34] Landis C, Back N, Homsher E, Tobacman LS (1999). Effects of tropomyosin internal deletions on thin filament function.. J Biol Chem.

[35] Hitchcock-DeGregori SE, Song Y, Moraczewska J (2001). Importance of internal regions and the overall length of tropomyosin for actin binding and regulatory function.. Biochemistry.

[36] Hitchcock-DeGregori SE, An Y (1996). Integral repeats and a continuous coiled coil are required for binding of striated muscle tropomyosin to the regulated actin filament.. J Biol Chem.

[37] Pato MD, Mak AS, Smillie LB (1981). Fragments of rabbit striated muscle alpha-tropomyosin. II. Binding to troponin-T.. J Biol Chem.

[38] Lehrer SS (1978). Effects of an interchain disulfide bond on tropomyosin structure: intrinsic fluorescence and circular dichroism studies.. J Mol Biol.

[39] Ueno H (1984). Local structural changes in tropomyosin detected by a trypsin-probe method.. Biochemistry.

[40] Sakuma A, Kimura-Sakiyama C, Onoue A, Shitaka Y, Kusakabe T (2006). The second half of the fourth period of tropomyosin is a key region for Ca(2+)-dependent regulation of striated muscle thin filaments.. Biochemistry.

[41] Lehrer SS (1975). Intramolecular crosslinking of tropomyosin via disulfide bond formation: evidence for chain register.. Proc Natl Acad Sci U S A.

[42] Sumida JP, Wu E, Lehrer SS (2008). Conserved Asp-137 imparts flexibility to tropomyosin and affects function.. J Biol Chem.

[43] Ishii Y, Lehrer SS (1985). Fluorescence studies of the conformation of pyrene-labeled tropomyosin: effects of F-actin and myosin subfragment 1.. Biochemistry.

[44] Palm T, Graboski S, Hitchcock-DeGregori SE, Greenfield NJ (2001). Disease-causing mutations in cardiac troponin T: identification of a critical tropomyosin-binding region.. Biophys J.

[45] Dyson HJ, Wright PE (2005). Intrinsically unstructured proteins and their functions.. Nat Rev Mol Cell Biol.

[46] Tompa P, Fuxreiter M, Oldfield CJ, Simon I, Dunker AK (2009). Close encounters of the third kind: disordered domains and the interactions of proteins.. Bioessays.

[47] Gazi AD, Bastaki M, Charova SN, Gkougkoulia EA, Kapellios EA (2008). Evidence for a coiled-coil interaction mode of disordered proteins from bacterial type III secretion systems.. J Biol Chem.

[48] Gazi AD, Charova SN, Panopoulos NJ, Kokkinidis M (2009). Coiled-coils in type III secretion systems: structural flexibility, disorder and biological implications.. Cell Microbiology.

[49] Greenfield NJ, Palm T, Hitchcock-DeGregori SE (2002). Structure and interactions of the carboxyl terminus of striated muscle alpha-tropomyosin: it is important to be flexible.. Biophys J.

[50] Hilser VJ, Thompson EB (2007). Intrinsic disorder as a mechanism to optimize allosteric coupling in proteins.. Proc Natl Acad Sci U S A.

[51] Heald RW, Hitchcock-DeGregori SE (1988). The structure of the amino terminus of tropomyosin is critical for binding to actin in the absence and presence of troponin.. J Biol Chem.

[52] Butters CA, Willadsen KA, Tobacman LS (1993). Cooperative interactions between adjacent troponin-tropomyosin complexes may be transmitted through the actin filament.. J Biol Chem.

[53] Ruiz-Opazo N, Nadal-Ginard B (1987). Alpha-tropomyosin gene organization. Alternative splicing of duplicated isotype-specific exons accounts for the production of smooth and striated muscle isoforms.. J Biol Chem.

[54] Studier FW, Rosenberg AH, Dunn JJ, Dubendorff JW (1990). Use of T7 RNA polymerase to direct expression of cloned genes.. Methods Enzymol.

[55] Hammell RL, Hitchcock-DeGregori SE (1996). Mapping the functional domains within the carboxyl terminus of alpha- tropomyosin encoded by the alternatively spliced ninth exon.. J Biol Chem.

[56] Hitchcock-DeGregori SE, Mandala S, Sachs GA (1982). Changes in actin lysine reactivities during polymerization detected using a competitive labeling method.. J Biol Chem.

[57] Edelhoch H (1967). Spectroscopic determination of tryptophan and tyrosine in proteins.. Biochemistry.

[58] Laemmli UK (1970). Cleavage of structural proteins during the assembly of the head of bacteriophage T4.. Nature.

[59] Urbancikova M, Hitchcock-DeGregori SE (1994). Requirement of amino-terminal modification for striated muscle alpha- tropomyosin function.. J Biol Chem.

[60] Whitby FG, Phillips GN (2000). Crystal structure of tropomyosin at 7 Angstroms resolution.. Proteins.

[61] Pettersen EF, Goddard TD, Huang CC, Couch GS, Greenblatt DM (2004). UCSF Chimera–a visualization system for exploratory research and analysis.. J Comput Chem.

[62] Kremneva E, Nikolaeva O, Maytum R, Arutyunyan AM, Kleimenov SY (2006). Thermal unfolding of smooth muscle and nonmuscle tropomyosin alpha-homodimers with alternatively spliced exons.. Febs J.

